# Computer modeling of spatial dynamics and primary genetic divergence for a population system in a ring areal

**DOI:** 10.18699/vjgb-25-115

**Published:** 2025-12

**Authors:** M.P. Kulakov, O.L. Zhdanova, E.Ya. Frisman

**Affiliations:** Institute for Complex Analysis of Regional Problems of the Far Eastern Branch of the Russian Academy of Sciences, Birobidzhan, Russia; Institute of Automation and Control Processes of the Far Eastern Branch of the Russian Academy of Sciences, Vladivostok, Russia; Institute for Complex Analysis of Regional Problems of the Far Eastern Branch of the Russian Academy of Sciences, Birobidzhan, Russia

**Keywords:** metapopulation, migration, spatiotemporal dynamics, mathematical modeling, genetic divergence, gene flow, hybrid zones, isolation, метапопуляция, миграция, пространственно-временная динамика, математическое моделирование, генетическая дивергенция, поток генов, гибридные зоны, изоляция

## Abstract

One of the main goals of modern evolutionary biology is to understand the mechanisms that lead to the initial differentiation (primary divergence) of populations into groups with genetic traits. This divergence requires reproductive isolation, which prevents or hinders contact and the exchange of genetic material between populations. This study explores the potential for isolation based not on obvious geographical barriers, population distance, or ecological specialization, but rather on hereditary mechanisms, such as gene drift and flow and selection against heterozygous individuals. To this end, we propose and investigate a dynamic discrete-time model that describes the dynamics of frequencies and numbers in a system of limited populations coupled by migrations. We consider a panmictic population with Mendelian inheritance rules, one-locus selection, and density-dependent factors limiting population growth. Individuals freely mate and randomly move around a one-dimensional ring-shaped habitat. The model was verified using data from an experiment on the box population system of Drosophila melanogaster performed by Yu.P. Altukhov et al. With rather simple assumptions, the model explains some mechanisms for the emergence and preservation of significant genetic differences between subpopulations (primary genetic divergence), accompanied by heterogeneity in allele frequencies and abundances within a homogeneous area. In this scenario, several large groups of genetically homogeneous subpopulations form and independently develop. Hybridization occurs at contact sites, and polymorphism is maintained through migration from genetically homogeneous nearby sites. It was found that only disruptive selection, directed against heterozygous individuals, can sustainably maintain such a spatial distribution. Under directional selection, divergence may occur for a short time as part of the transitional evolutionary process towards the best-adapted genotype. Because of the reduced adaptability of heterozygous (hybrid) individuals and low growth rates in these sites (hybrid zones), gene flow between adjacent sites with opposite genotypes (phenotypes) is significantly impeded. As a result, the hybrid zones can become effective geographical barriers that prevent the genetic flow between coupled subpopulations.

## Abstract (ru)

Одна из ключевых задач современной эволюционной биологии – изучение процессов, приводящих к первичному разделению (дивергенции) популяций на различающиеся генотипами группы особей. Для дивергенции очевидно необходима репродуктивная изоляция, которая делает невозможным контакт особей или существенно затрудняет обмен генетической информацией между популяциями. Настоящее исследование изучает возможность изоляции, в основе которой лежат не очевидные географические барьеры, удаленность популяций или экологическая специализация, а лишь наследственные механизмы, дрейф и поток генов, а также отбор против гетерозигот. Для этого предложена и исследована динамическая модель с дискретным временем, которая описывает динамику частот и численностей в системе миграционно связанных лимитированных популяций. Рассматривается панмиктичная популяция с менделевскими правилами наследования, монолокусным отбором, действием плотностно-зависимых факторов, лимитирующих рост численности. Особи свободно скрещиваются и перемещаются вдоль одномерного кольцевого ареала. Модель верифицирована с использованием данных эксперимента над ящичной системой популяций дрозофил Drosophila melanogaster, проведенного под руководством Ю.П. Алтухова. При достаточно простых предположениях модель описывает некоторые механизмы возникновения и сохранения на однородном ареале существенных генетических различий (первичной генетической дивергенции), сопровождаемых неоднородностью в частотах аллелей и численностях. В этом случае формируется несколько больших групп генетически однородных субпопуляций, развивающихся независимо. В местах их контакта активно идет гибридизация, а полиморфизм сохраняется за счет миграции с сопредельных однородных участков. Обнаружено, что устойчиво поддерживать такое пространственное распределение может только дизруптивный (разрывающий) отбор, направленный против гетерозигот. При движущем отборе дивергенция существует непродолжительное время, как часть переходного процесса. За счет пониженной приспособленности гетерозигот (гибридов) и низкой скорости роста на этих участках (зонах гибридизации) существенно затрудняется обмен генами между смежными участками с противоположными гомозиготными генотипами (фенотипами). В результате зоны гибридизации выполняют функцию географического барьера, который фактически останавливает обмен генов между разными группами в случае смежной симпатрии.

## Introduction

Genetic divergence cannot occur without effective mechanisms of reproductive isolation and stopping the gene flow between populations. This can be caused by large distances between populations (allopatry), which cannot be overcome during the lifetime of individuals, or by geographical barriers that prevent the transfer of genes. However, even if populations of the same species live in the same or adjacent areas (sympatry or parapatry) they can differ significantly in their traits. Although individuals from these populations can interact and produce viable, fertile hybrids, there is no blurring of parental traits. Several mechanisms support the reproductive isolation and the divergence between different forms, including selection against hybrids, which often have lower fitness than parental populations.

There are sufficient examples of reproductive isolation, where different subpopulations have accumulated sufficient differences even when they live sympatrically and have developed effective measures to prevent hybridization. For instance, recognition signals related to phonetic features and used in mating behavior contribute to the stabilization of extreme forms of a characteristic. Thus, the mating calls of certain frog species (such as Microhyla carolinensis and M. olivacea, Litoria verreauxii and L. v. alpina) differ greatly in the contact zone where their ranges overlap, but do not differ significantly in areas where they do not occur together (Blair, 1955a; Littlejohn, 1965; Smith et al., 2003). In addition, the body sizes of different frog forms differ greatly in the contact zone, which complicates the mating process (Blair, 1955b).

Prezygotic isolation of sympatric forms of the same species or subspecies is often followed by ecological specialization, which does not prevent copulatory behavior between individuals with different traits and their hybridization, but only makes it unlikely. For example, the periods of sexual activity for two species of Rhagoletis pomonella are determined by the time of fruiting of the trees they were born on and lay their eggs on – hawthorn and apple (Filchak et al., 2000). These two races of flies of R. pomonella differ in their sensory processing of key fruit odors: while some individuals are attracted to apple and avoid hawthorns, others choose hawthorn and avoid apples, which significantly hinders their contact (Tait et al., 2021). The mating preferences of hybrids are not entirely clear. However, when two races of R. pomonella are interbred in the laboratory, a lower conception rate is recorded (Yee, Goughnour, 2011), which signals some selection against hybrids and persistent divergence in nature caused by specialization of flies

There are a few examples of hybridization where it does not have obvious negative effects, such as reduced fitness or a catastrophic decline in the reproductive success of hybrids (heterozygotes). For example, intraspecific variability in some birds is often expressed as differences in plumage coloration. At the same time, there is a clear divergence in traits between different parts of a large range, and stable hybrid zones exist over long periods of time in areas where the ranges overlap. The populations of the carrion crow and hooded crow (Corvus corone and C. cornix) are well known in Siberian (between the Ob and Yenisei rivers) and European hybrid zones (Haring et al., 2012; Poelstra et al., 2014; Kryukov, 2019; Blinov, Zheleznova, 2020), or northern flicker hybrid zone (Colaptes auratus cafer and C. a. auratus) in USA (Aguillon, Rohwer, 2022). Another example is the hybridization of the great tit (Parus major) and Japanese tit (P. minor) in the Amur region (Kapitonova et al., 2012).

A genetic mechanism supporting isolation based on innate mating preferences has been identified in crows: they prefer to choose partners who are similar to themselves rather than exotic individuals. The process of forming phenotypes in carrion and hooded crows is linked to chromosomal inversion, which affects both feather coloration and the visual perception of feather colors, as well as certain aspects of reproductive behavior (Poelstra et al., 2014). However, in areas where hybridization occurs, which apparently arises simultaneously with different colorations, mating preferences turn out to be more diverse and complete isolation does not occur. This is because the inverted chromosome region of the hooded crow is inherited in its entirety and does not recombine with the homologous regions of the carrion crow.

One simple model for studying genetic divergence is a linear chain or ring of partially isolated subpopulations that exchange genes. The studies on such models show that gene flow between subpopulations coupled by migration can lead to stable geographic variability of a trait and the maintenance of hybrid zones only with disruptive selection. With directional selection, stable divergence is impossible and can only occur as part of a transition process under special initial conditions (Bazykin, 1972; Frisman, 1986; Yeaman, Otto, 2011; Láruson, Reed, 2016). For chains of connected populations with different topologies, it has been found that divergence occurs more often in linear chains and rings, and less often in fully connected networks (with global connectivity) (Láruson, Reed, 2016; Sundqvist et al., 2016).

At the same time, for many natural populations with significant divergence in characteristics and sometimes with known isolating mechanisms, it can be difficult to identify a specific adaptive trait that disruptive selection acts upon. This may be due to hidden traits, such as innate immune factors or the major histocompatibility complex, which are not directly related to an external trait that we currently observe in individuals, such as feather coloration in birds, skin or coat patterns, beak shape and size, or behavioral characteristics. The observed spatial distribution of a trait does not directly indicate the causes or type of selection that led to this divergence in the past. However, it can be successfully linked to the observed trait and serve as an indicator or marker of fitness, particularly for species with wide ranges, heterogeneous environmental conditions, significant divergence, and a high degree of polymorphism (Orsini et al., 2008; Murphy et al., 2010).

This work is part of a series of studies investigating the basic mechanisms of primary genetic divergence in systems of panmictic populations of diploid organisms coupled by migration and selection directed against heterozygotes (Zhdanova, Frisman, 2023; Kulakov, Frisman, 2025). We propose a dynamic discrete-time model that takes into account the action of density-dependent factors limiting population growth, genetic drift (through certain perturbations of initial conditions), natural selection, and migration of individuals between adjacent sites. The model is verified based on data from laboratory experiments with box populations of Drosophila (Drosophila melanogaster) conducted under the supervision of Yu.P. Altukhov, which showed significant divergence in allele structure at the α-glycerophosphate dehydrogenase (α-Gdph) locus between groups of adjacent boxes (Altukhov et al., 1979; Altukhov, Bernashevskaya, 1981; Altukhov, 2003).

In this article, we analyze the processes of selection and migration (gene flow) that form and maintain the heterogeneous spatial distribution of allele frequencies, based on multiple computer simulations of a model. We investigate the role of hybrid zones with high proportions of heterozygous individuals in the α-Gdph gene and demonstrate that these zones separate monomorphic groups of boxes apart and do not allow the most adapted genotype to spread throughout the entire ring area.

## Materials and methods

The study is based on an original mathematical model – a system of coupled nonlinear maps (discrete-time equations) that describes the dynamics of genotype frequencies and subpopulation abundances. The migration of individuals and gene flow between subpopulations are described using a migration matrix with random coefficients. We use the MT19937 random number generator (Matsumoto et al., 1998), available in the GSL numerical computation library. This generator has an extremely long period (~106,000) and low correlation, passing most statistical tests for randomness in its pseudo-random number sequences

To validate the model, we use data from an experiment on the D. melanogaster ring system, conducted by a team led by Yu.P. Altukhov. The data consist of allele frequencies at the locus encoding the α-Gdph enzyme, as well as the numbers of flies in each box at different stages of the experiment (Altukhov, 2003). We estimate model parameters using the least squares method.

Numerical experiments are conducted with the author’s software package, including the computer implementation of a mathematical model, visualization of the results, and analysis of dynamic regimes.

## Model of local population

We consider a population of diploid organisms where between two adjacent generations, the following sequence of elementary population processes occurs: zygote formation from gametes, natural selection on zygotes (individuals), migration (dispersal) between adjacent subpopulations, and production of new gametes. We focus on populations in which the adaptive diversity is determined by a single locus with two alleles (A and a), which are inherited co-dominantly. The phenotype of individuals is strictly determined by their genotype. The population is panmictic, and Mendelian inheritance rules apply. This means that the population contains individuals with genotypes AA, Aa, and aa. At time t, these genotypes have abundances N1(t), N2(t), and N3(t), respectively, and frequencies q1(t) = N1(t) / N(t), q2(t) = N2(t) / N(t), and q3(t) = N3(t) / N(t) (where N(t) = N1(t) + N2(t) + N3(t) is the total population size).

Let us assume that the genotypes differ in their reproductive abilities, which is expressed by differences in gamete production rates or individual survival rates. Denote the intensity of gamete production for individuals with genotypes AA, Aa, and aa as gAA, gAa and gaa, respectively, taking into account the death of some gametes before they combine into zygotes in the next generation. Additionally, let WAA, WAa and Waa represent the proportion of zygotes (or individuals) with the corresponding genotype that survive the natural selection and have the ability to migrate (disperse).

In cases where gamete production intensity does not depend on parental genotypes, i. e., gAA = gAa = gaa = g, the equations for genotype frequencies in a local panmictic population can be expressed as:

**Formula. 1.**
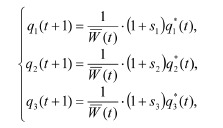
Formula. 1

where q 1*(t) = (q1(t) + q2(t)/2)2, q 2*(t) = 2(q1(t) + q2(t)/2)(q3(t) + q2(t)/2), q 3*(t) = (q3(t) + q2(t)/2)2 are the genotype frequencies immediately after gametes combine into zygotes, but before selection and migration of individuals (Zhdanova, Frisman, 2023; Kulakov, Frisman, 2025). The parameter sk is the selection coefficient for zygotes with the corresponding genotype, which links the fitness Wk of each genotype and the gamete production rate gk as follows: 1 + sk = gWk (k =AA, Aa, aa). In system (1), the normalization factor

**Formula. 2.**
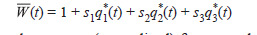
Formula. 2

is equal to the average (generalized) fitness, and its value determines the population growth rate. If there are no factors limiting the growth, the population size changes according to the following equation:

**Formula. 3.**
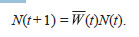
Formula. 3

The number of individuals with each genotype is determined by ratios: Nk(t + 1) = qk(t + 1)N(t + 1) = (1 + sk) q k*(t)N(t + 1) (k=AA, Aa, aa).

Of all the types of genetic selection determined by values s1, s2, and s3, disruptive selection is the most interesting (s2 < s1 and s2 < s3), as system (1) demonstrates bistability. Early studies show that this type of selection is responsible for the emergence and fixation of genetic differences in different parts of a homogeneous area, even when environmental and other factors are not considered.

At the same time, on a large temporal scale, the growth of actual evolving populations is limited by environmental factors. This growth limitation can be described by a nonlinear dependence of selection and gamete production parameters on the abundance of genotypes or the total population density in model (1)–(3). It is easy to show that if the rates of gamete production are equal for all genotypes, then there is no difference between the limiting gamete production rate (g) and the intensity of selection (Wij) in case of competition for a common resource. Therefore, without loss of generality, we can assume that:

**Formula. 4.**
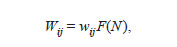
Formula. 4

where wij is the maximum proportion of individuals with genotype ij (AA, Aa, or aa) that survive after natural selection under minimal competition (at low density), F is the function that describes the effect of density-dependent growth limitation, and N is the total population size. Considering (4), the frequency dynamics equations (1) will not change their form, except for replacing Wij with wij and gWij with 1 + sk , while the population equations (3) will have a nonlinear dependency on density:

**Formula. 5.**
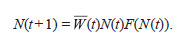
Formula. 5

In populations of diploid organisms, exchange of gametes often requires contact between individuals. The probability of this decreases significantly at low densities, i. e., there is a direct correlation between the average individual fitness and the population density – the Allee effect (Allee, 1958). As a result, when the population size falls below a certain critical value N0, population growth becomes impossible and effective natural selection ceases to operate. Instead, only genetic drift determines the evolutionary trajectory of the population. Therefore, to describe these density-dependent limiting factors, we can use a function of the following form:

**Formula. 6.**
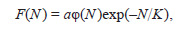
Formula. 6

where φ(N ) is a sigmoid function equal to:

**Formula. 7.**
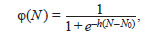
Formula. 7

with parameter h ≥ 2, which defines the slope angle of the sigmoid at point N0. The value of N0 determines the minimum population size required for simple reproduction (1:1). The parameter K defines the ecological capacity of the habitat, and a defines the average number of offspring per individual with an average fitness of 1. These two parameters determine the steady-state (equilibrium) population size N ≈ K ln(a ). Using (7), we can rewrite the equation (5) for population dynamics as follows:

**Formula. 8.**
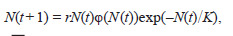
Formula. 8

where r = a (t) is the total reproductive capacity of all genotypes.

When r > 1, equation (8) has three fixed points [N (t + 1) = = N (t)]: 0, N0 and N ≈ K ln(a ). If N < N0, the number of surviving offspring N (t + 1) is less than the number of their ancestors N (t), and the population inevitably declines, which corresponds to a strong Allee effect. If N0 < N < N and r > 1, there are enough breeders and the population size increases. With N > N, the population size exceeds the carrying capacity of the habitat, and the population abundance falls to a steady-state of N

Let us now consider populations that are coupled by migration and evolve in the way described above.

## Dynamic model with gene flow

**Fig. 1.**
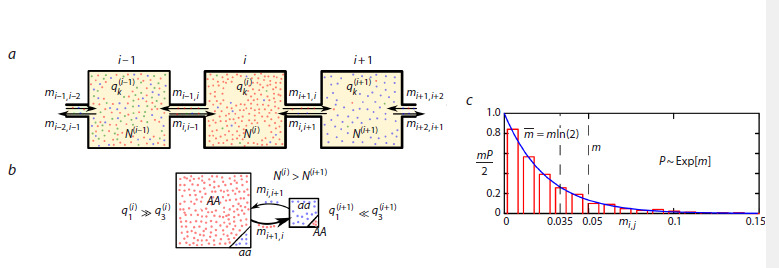
a, Scheme of the population system – boxes coupled by narrow migration corridors. b, Illustration showing that gene flow between populations of different sizes can significantly change the genotype in a small population, but has no effect on a large population. c, The probability density of an exponentially distributed random value of the migration coefficient mi, j .

One method for studying the dynamics and evolution of dispersed population systems (metapopulations) is to conduct laboratory experiments using populations in boxes that are connected by narrow corridors. In these experiments, environmental conditions, growth parameters, selection, and migration can be carefully controlled. Typically, the connected boxes (chambers) form closed chains of subpopulations that exchange a small number of individuals (Fig. 1a). These population systems are often constructed in laboratory settings, for example, for D. melanogaster (Altukhov et al., 1979; Altukhov, Bernashevskaya, 1981; Dey, Joshi, 2006), or Escherichia coli (Keymer et al., 2006).

Consider a system of n boxes, or subpopulations, and each box is numbered from 1 to n (Fig. 1a). Let 0 ≤ mi, j <1 denote the proportion of individuals from the total population size that move from box j to box i (mi, j is the migration coefficient). The emigrants consist of individuals with three studied genotypes, so it is true that mi, j N ( j) = mi, j q( j) AA N ( j) + mi, j q( j) Aa N ( j) + + mi, j q( j) aa N ( j

Then, for a system of subpopulations coupled by migration, the equations for frequency dynamics (1) and abundance dynamics (8) take the following forms

**Formula. 9.**
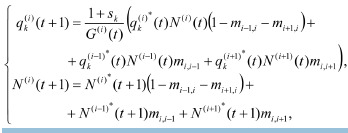
Formula. 9

where k = 1, 2, 3 are the numbers of the groups of individuals with the genotypes AA, Aa, and aa, respectively, q(i)* k are the frequencies before migration, and N (i)*(t + 1) = = a (i)(t) N (i)(t) F(N (i)(t)) is the abundance of the ith subpopulation after selection but before migration. The normalization coefficient G is equal to:

**Formula. 10.**
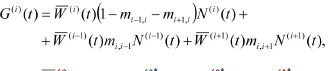
Formula. 10

where (i)(t) = 1 + s1q(i)* 1 (t) + s2q(i)* 2 (t) + s3q(i)* 3 (t). To close the chain of subpopulations into a ring, we assume that the 1st box is connected to the 2nd and nth, the nth to the (n–1)th and 1st, i. e., the following mapping applies to the site number: i→i mod n. In system (9), the factor (1 – mi–1,i – mi+1,i) is the proportion of individuals that stayed in the ith box after migrating to the two neighboring boxes; mi,i–1 and mi,i+1 are the proportions of individuals from (i–1) and (i+1)-subpopulations that migrated to the ith box

Equations (9) demonstrate that the intensity of gene flow from each subpopulation is not only dependent on the frequencies of genotypes within the native site, as was the case for the local population, but also on the absolute number of individuals. This is clearly evident from the assumption that migrants consist of individuals with all three possible genotypes. Therefore, the flow of migrants from a small population consisting, for example, solely of aa homozygotes, has a minimal impact on a larger population consisting mainly of AA homozygotes (Fig. 1b). Conversely, the flow from a larger population can quickly change the frequencies even at a low migration rate. Note that, in some cases, this mechanism clearly violates the assumption of panmixia at the scale of the entire metapopulation, as changes in the frequency of non-comparable subpopulations are determined more by the genetic structure of immigrants than by random mating, genetic drift, or natural selection.

The flow of genes and individuals between subpopulations can be either completely deterministic or random. In the first case, the number and genetic structure of migrants depend on factors such as population density at the source and sink sites, or external environmental factors like food (taxis) and energy flows (phototaxis). In the second case, both the direction and proportion of migrants vary randomly from generation to generation, without any clear pattern.

Below, we will only consider random migration. To describe this, we do the following. For each season number t, we randomly select two migration coefficients mi–1,i and mi+1,i , which are equal to the proportions of individuals that leave the ith site and migrate to adjacent sites. We ignore the possibility of more distant dispersal. Each pair of values mi–1,i and mi+1,i will be generated independently using an exponentially distributed random variable generator with an expected value of m/2 and a median of mln(2).

Figure 1c shows a histogram of the distribution of 200 replicates, each consisting of 30 pairs of independent random values for migration coefficients (n = 30 and m = 0.05), along with the graph of the theoretical probability density function. Both curves are scaled to the same distribution parameter λ = 2m–1. This value corresponds to a situation where approximately half of all migration coefficients are less than or equal to mln(2) ≈ 0.035, and their average is m = m/2 = 0.025

Next, we consider the dynamic regimes in the system (9)– (10) with random migration, using parameter values obtained from experimental data.

## Model verification

There are two ways to verify the model and search for conditions of primary genetic divergence. First, we can perform a series of simulations to ensure that the system (9) generates regimes corresponding to genetic divergence with only reduced heterozygote fitness. Secondly, we need to compare the results of simulations with the empirical data. However, this can be challenging, as despite all the available research and data, most natural populations with clear divergence in traits across space are initially highly heterogeneous

The ideal solution may involve using data from a carefully designed animal experiment. In the mentioned experiment, conducted under the supervision of Yu.P. Altukhov, evolutionary processes were studied in a system consisting of 30 boxes connected by narrow tubes and inhabited by D. melanogaster flies (Altukhov et al., 1979; Altukhov, Bernashevskaya, 1981). The randomness of migration was provided by uniform environmental conditions (lighting and food) and random rotation of the ring system of connected boxes. During the experiment, the spatial distribution and abundance dynamics, as well as the frequency of alleles at the autosomal esterase-6 (Est-6) and α-glycerophosphate dehydrogenase (α-Gdph) loci, were analyzed. By the 60th generation, a clear and stable differentiation of allele distribution at the α-Gdph locus formed between groups of adjacent boxes

Some parameters are immediately known from the description of the original experiment, such as the migration coefficient (m ≈ 0.03) and the number of boxes (n = 30). Initially, a few heterozygous individuals for the considered loci (150 pairs, from 1 to 37 in each box) were placed in the boxes, i. e. q(i) 2 (0) = 1. At the same time, a large panmictic population was established, which was similar in size and initial frequency to the system of connected boxes. Based on the frequency dynamics of the A allele at the α-Gdph locus in a large population, we can easily estimate the selection parameters sk (see the Table). As a basis for our study, we used the values of sk derived from earlier work (Zhdanova, Frisman, 2023), where they were obtained using a one-dimensional equation for the frequency of allele A of the α-Gdph locus. The pattern of change in the frequency of allele A in the experiment closely matches the typical solution of model (1), with disruptive selection (s2 < s1 and s2 < s3) rather than directional selection (s1 > s2 > s3 or s3 > s2 > s1).

Based on the initial conditions (N(i)(0) = 1…37, Σ N (i)(0) = = 300), the population growth pattern, and the limiting number of individuals in each box (N(i) ≈ 135), as well as in the local panmictic population, we can easily calculate the parameters for population growth, including values of a, h, N0 and K, which are shown in the Table

**Table 1.**
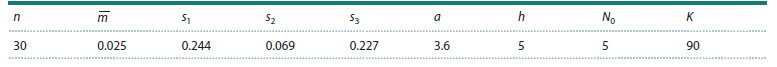
Values of parameters for model (9)

The average migration coefficient m = 0.025 in the Table and the median value of mln(2) ≈ 0.035 indicate that in most cases, the number of migrants does not exceed 4–5 individuals, which is similar to the results of the original experiment.

The greatest difficulty in verifying the model (9) involves selecting initial distributions of allele frequencies and abundances that yield final distributions similar to those presented in Chapter 4 of the book (Altukhov, 2003). In order to select initial conditions, we generate a set of initial frequencies and abundances using a feature of the experiment: individuals of the same sex are randomly included in some boxes and do not produce offspring. To describe this, let us create a vector of random numbers as follows: N (i)(0) ~ U [0, 37], so that Σ N (i) (0) ≈ 300, and let some boxes be initially empty (N (i)(0) = 0). As a result, since 0 ≤ N (i)(0) < N0 (lower than the effective number of breeders), in subsequent generations, the boxes will still remain empty and will be recolonized by migrants from neighboring boxes, the genetic structure of which may already differ significantly from the original one due to random genetic drift and selection. However, there may not be enough migrants to effectively sustain the subpopulation, and the box may remain empty for several generations.

Because the initial numbers in all boxes are below the effective population size (Ne), the natural selection is not effective, and we cannot ignore the effect of random genetic drift. The authors of the outlined experiment assumed Ne ≈ 50. This means that after the 2nd or 3rd generation, the effect of deterministic selection processes begins to dominate over random processes that change allele frequencies. It would be difficult to directly describe genetic drift in the model (9) without significant modification or transitioning to a simulation model. Instead, we “simulate” the result of genetic drift by using the most likely initial frequency distribution, which is typically formed in model (1). With disruptive selection (sk values from the Table), system (1) predicts that the frequencies of offspring genotypes in the 2nd and 3rd generations from completely heterozygous ancestors (with q2(0) = 1) will be approximately q1 ≈ 0.27, q2 ≈ 0.46 and q3 ≈ 0.27. We can assume that, for the first few generations, genetic drift will randomly shift the frequencies away from their initial values while the population sizes remain below the effective population size Ne. As a result, the observed genetic divergence in the system of coupled populations can be equally explained by the initial differences in both population sizes and frequencies, caused by the initial genetic drift prior to reaching the effective size in each subpopulation.

To fit the initial frequencies, we generate two independent vectors of random numbers: q(i) 1 (0) ~ U [0,1] and q(i) 2 (0) ~ U [0,1] (q(i) 3 (0) = 1 – (q(i) 1 (0) + q(i) 2 (0))), and estimate how much the “true” initial frequencies may vary from the theoretical values of 0.27, 0.46, and 0.27 due to drift, so that after 50–60 generations, model (9) approximately describes the real distribution of allele A frequencies at the α-Gdph locus. After examining 300,000 randomly selected initial frequencies and abundances, we found that only about 100 replicas most accurately describe the actual distribution, with the following distribution of initial frequencies:

**Formula. 11.**
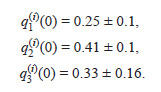
Formula. 11

**Fig. 2.**
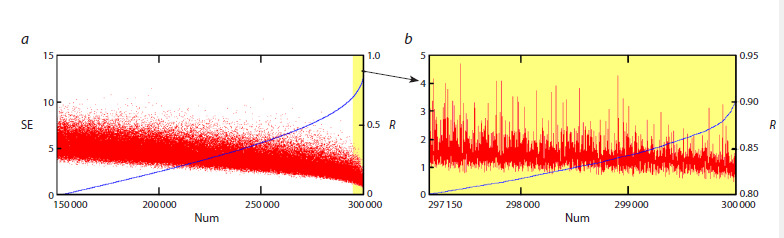
Squared errors SE and correlation coefficients R for 300,000 initial conditions are ranked in order of increasing R. The Num is the “number” of initial conditions.

This shows that we obtain a slightly lower frequency of heterozygotes and a shift towards homozygosity with the aa genotype than those predicted by model (1). Note that the experimental data also showed a slight shift in the average frequency of allele A towards allele a in the 5th generation, despite the lower fitness of s3. Therefore, it would be reasonable to choose initial frequencies within these ranges. From a new set of 300,000 initial conditions of type (11), about 3,000 describe the actual frequency distribution quite well (Fig. 2). To assess the quality of the approximation, we used the correlation coefficient R between the actual and model frequency distributions of allele A at the α-Gdph locus in generation t, as well as the squared error SE:

**Formula. SE.**
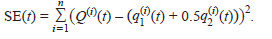
Formula. SE

## Simulation results

We now consider the verification of equations (9) and analyze the mechanisms leading to stable genetic divergence

**Fig. 3.**
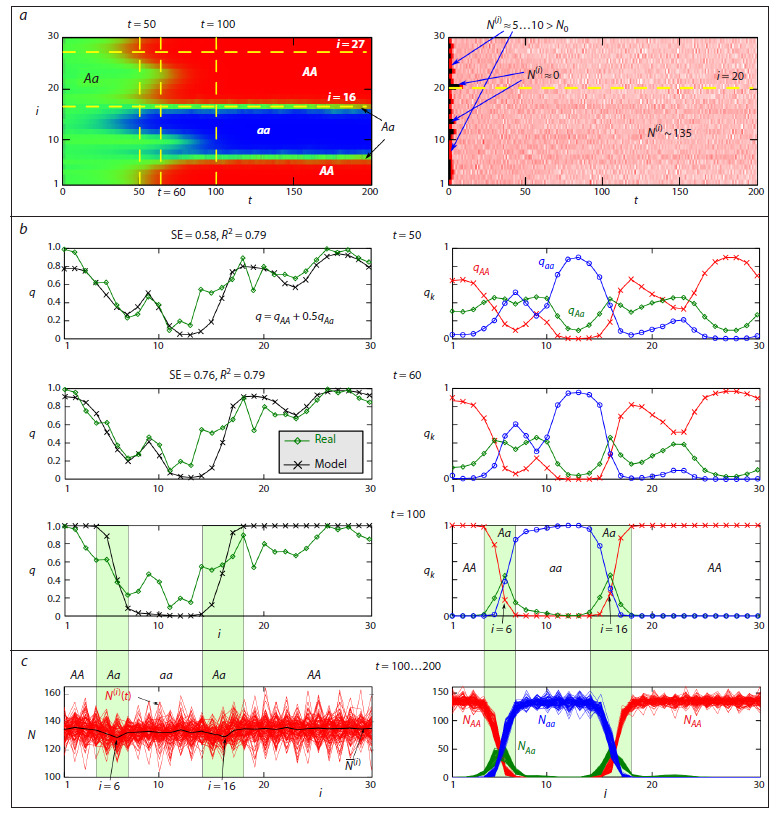
a, Spatiotemporal dynamics of genotype frequencies and population sizes in the system of migration-coupled populations described by model (9). b, Modeled and observed frequency (q) distributions of the allele A at the α-Gdph locus and the frequency (qk) of zygotes at the 50th, 60th, and 100th generations. c, The distribution of the total population size across the area (left), along with its components represented by the numbers of individuals with genotypes AA, Aa и aa.

Figure 3a shows two diagrams of the spatiotemporal dynamics in system (9) for the parameter values from the Table, using the most favorable initial conditions (Fig. 2b).

In the first diagram, the pixel color encodes the predominant genotype at site i and time t; in the second diagram, it encodes the population size. Figure 3a shows that at the initial stages, all subpopulations are polymorphic and contain all three genotypes (shown in green). Over time, driven by selection and the dispersal of individuals within the distributed system, an equilibrium state is established. This state corresponds to a stable genetic divergence that persists for a long time (including for t >> 200). In one part of the boxes, only individuals with the AA genotype (red) are present; in another, only those with the aa genotype (blue) are found; polymorphic subpopulations with a high frequency of heterozygotes (green) are located between them. In the diagram, the subpopulation numbered i = 16, along with its neighbors, maintains polymorphism for t >> 200. The second diagram shows changes in population size, where pink corresponds to the maximum values (~135) and black to the minimum ones. This diagram reveals several boxes that were initially empty, demonstrating that their location does not correlate with the final distribution of genotypes

As can be seen from Figure 3b, model (9) describes the observed frequency distribution quite well. However, in all simulation runs (i. e., replicas with varied migration coefficients, mi, j), the distribution similar to that observed in the Drosophila experiments emerges slightly earlier – around the 50th generation rather than the 60th. This discrepancy could be attributed to inaccurately estimated growth parameters since the equations (9) seem to describe a slightly faster population growth and evolutionary rate than is observed in reality. Alternatively, genetic drift processes, which were simulated using random initial frequencies, may have prevailed over selection for a longer period in the real experiment than we assumed (e. g., for 2–3 generations until the population size reached an effective Ne ≈ 50). However, there is another probable explanation. In the experiments with D. melanogaster, the sex and age composition of all subpopulations was artificially maintained to prevent generation overlap. Specifically, all adult individuals were removed from the boxes after the females laid eggs. However, the sex ratio varied considerably between boxes throughout the experiment. Some boxes exhibited a significant deficit of females, while others had a pronounced shortage of males. Consequently, not all females were able to produce offspring before the removal time, and some males fertilized multiple females. This violation of panmixia likely skewed the data, as each complete removal event set back the evolutionary process slightly. These complex processes are not fully captured by the relatively simple model (9), which is why it predicts a slightly faster rate of evolution.

In Figure 3c, the final 100 distributions (for t = 100…200) of the total population size for each genotype are superimposed. The figure shows that, due to fluctuations in the number of migrants, the population size in different boxes undergoes irregular, non-synchronous oscillations. Furthermore, it is evident that the polymorphic subpopulations (i = 6 and 16) have a lower average abundance (N(i)) than the surrounding monomorphic subpopulations, which is consistent with the significant frequency of heterozygotes in these populations.

**Fig. 4.**
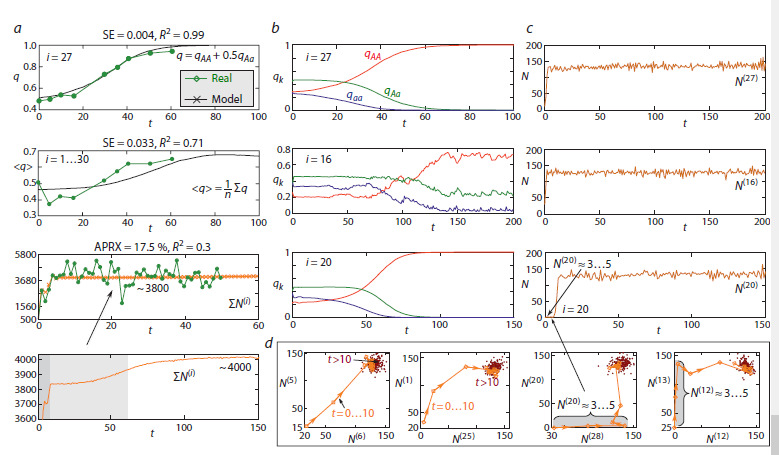
a, Modeled and observed dynamics of the frequency q of allele A at the α-Gdph locus and the total number of populations of D. melanogaster in the box system. SE is the squared error, R2 is the coefficient of determination, and APRX is the approximation error. Model dynamics of genotype frequencies (b) and population sizes (c) of the subpopulations highlighted in Fig. 3a. d, Phase portraits illustrating the group dynamics of the two subpopulations; the light brown color denotes the stage of rapid box colonization, and brown indicates the transition to the maximum population size.

As shown in the first diagram of Figure 3a, the subpopulations evolve at different rates. This rate is determined by how close the initial population size of a subpopulation is to the effective size (Ne) and how close its initial allele frequency is to its final state (q = 1 or 0). For instance, the diagram highlights box i = 27, where the frequency of allele A was among the first to reach fixation (q = 1). Notably, this subpopulation evolves similarly to a large panmictic population (the first graph in Fig. 4a). Other subpopulations, as a rule, evolve more slowly.

Figure 4 demonstrates the correlation between the dynamics of allele frequencies and population sizes predicted by model (9) and the actual experimental data. Figure 4a shows that the modeled and experimentally observed average frequency of allele A across all 30 boxes follow a similar trend, stabilizing at a value of q ≈ 0.65. The discrepancy between the modeled and observed average frequency at time point t = 5 can be explained by the fact that model (9) does not directly account for genetic drift, which occurred in the experimental population; instead, its effect is simulated solely through random perturbations of the frequency in the polymorphic population.The third graph, Figure 4a, shows the observed and modeled total population sizes for the system of 30 subpopulations. The fourth graph (Fig. 4a) shows that the transition to the maximum population size proceeds through three stages: explosive growth over 2–3 generations from a small number of founders; reaching a quasi-stationary level with a total size of approximately Σ N (i) ~ 3800 individuals, at which point there is already a distinct differentiation of genotypes by box groups, but the system still remains sufficiently polymorphic (Fig. 3b at t = 50); and a transition to the final distribution (Fig. 3b at t = 100) and the maximum total population size of approximately 4,000 individuals. As can be seen, model (9) describes only the general trends of population growth, which is explained by the fact that its behavior is, in principle, the only possible type of dynamics at r = a < e2 ≈ 7.38. Furthermore, equation (8), which describes the dynamics of a local population, does not account for sex and age structure or many other factors that undoubtedly caused irregular fluctuations in the experimental populations. More importantly, model (9) describes only the reproductive core of the population system – females and an equal number of males – and does not consider the fact that some males could have remained single and constituted the majority of migrants. As a result, the modeled population size is lower than the actual observed size.

At the same time, the modeled dynamics of the total population size, Σ N (i), result from non-synchronous fluctuations of each subpopulation around a stationary value of approximately 135 individuals per box (Fig. 4c, d). Summing these values smooths out all differences in the sizes of the subpopulations. Despite heterogeneities in the initial distributions of individuals, population growth in the first 5 generations – driven by increased fitness – occurs synchronously in almost all boxes (the first and second panels in Fig. 4d). The exception are boxes that were initially empty or had an insufficient number of breeders (the third and fourth in Fig. 4d). For these boxes, a non-zero population size of approximately 3–5 individuals is maintained solely by migrants. In all other boxes, the numbers slowly reach their maximum values and fluctuate around them (dark dots in Fig. 4d).

We now consider the mechanisms that could generate and maintain the observed spatial divergence in allelic composition within this experimental population system.

## Analysis of migration flows

One of the reasons for the observed differentiation between the subpopulations is revealed by the small declines in population size in boxes i = 6 and i = 16, where polymorphism was maintained (boxes designated as Aa in Fig. 3). These declines become apparent only in the final distribution, as these boxes are surrounded by subpopulations with opposite genotypes and have a large population number. However, the presence of such subpopulations indicates only the possible mechanisms for maintaining divergence, rather than the reasons of its initial occurrence. These boxes can be considered as the hybrid zones, the allelic composition of which is maintained solely through migration and gene flow from sites inhabited by individuals with fixed opposite genotypes.

**Fig. 5.**
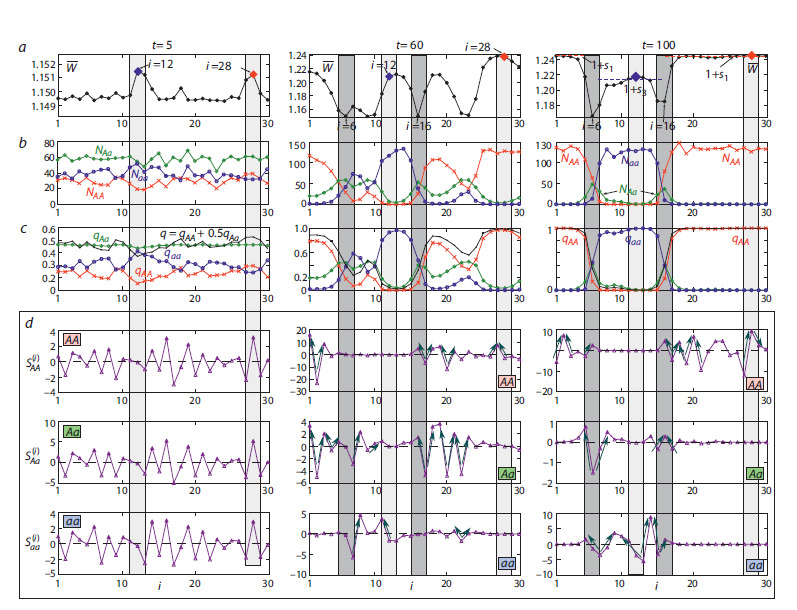
Distribution of the average fitness values for each subpopulation before migration (a), population sizes (b), and frequencies (c) of the AA, Aa, and aa genotypes. d, Distribution of the migration balance values for each site. The graphs highlight the areas where groups with the AA (i = 28) and aa (i = 12) genotypes are formed, as well as areas with active hybridization of individuals (i = 6 and i = 16). The arrows on the balance charts indicate the flow directions of individuals with the corresponding genotypes.

To study the mechanisms of the formation and maintenance of divergence, we will consider changes in the average fitness in each box (i) (Fig. 5a), the numbers of individuals of each genotype N (i) k (Fig. 5b), and allele frequencies q(i) k (Fig. 5c) over time. We will also assess the contribution of migration to the process of natural selection and the transition to the final frequency distribution. The migration balance of individuals with genotype k (k = AA, Aa, or aa) in the subpopulation i will be calculated using the following formula:

**Formula. 12.**
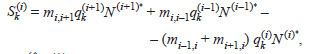
Formula. 12

where q(i) k N (i)* represents the number of individuals with genotype k after selection, but before migration. This value is equal to the difference between the number of arrivals (the first two terms) at the site with index i and the number of departures (the third term) of individuals. The value of S indicates whether the size of the subpopulation with index i has increased (S > 0) or decreased (S < 0) due to migration (Fig. 5d). By comparing these three values, we can easily determine the directions of migration (arrows in Fig. 5d).

where q(i) k N (i)* represents the number of individuals with genotype k after selection, but before migration. This value is equal to the difference between the number of arrivals (the first two terms) at the site with index i and the number of departures (the third term) of individuals. The value of S indicates whether the size of the subpopulation with index i has increased (S > 0) or decreased (S < 0) due to migration (Fig. 5d). By comparing these three values, we can easily determine the directions of migration (arrows in Fig. 5d).

When selecting the initial conditions, it was found that the experimentally observed frequency distribution in model (9) occurs when the initial frequencies are shifted toward the prevalence of homozygotes with the aa genotype. Note that the AA and aa genotypes differ in fitness by approximately 11 %. This means that for the most adapted AA genotype to become fixed, it must overcome this fitness threshold for a small proportion of subpopulations. However, a rarer set of circumstances is required for the less adapted aa genotype to avoid complete displacement, allowing both traits to be maintained.

Figure 5a shows that after a period of rapid growth until the 5th generation, two sites are distinguished, numbered i = 12 and i = 28, in which the frequency distribution yields the highest values of both average fitness (i) and total reproductive potential a (i) among all others. Although this difference is small (1 % for aa and 0.7 % for AA), it proves sufficient to initiate the separation of individuals of the same genotype near these boxes. This likely required a frequency shift in more than one site. Figures 5b and 5c show the distributions of population sizes and genotype frequencies, respectively. It can be observed that near site i = 12 at t = 5, there are at least six boxes with an increased number of aa homozygotes (and q < 0.5) relative to their surroundings. This implies that the flow of migrants from this region for any random mi, j is primarily represented by this genotype, which promotes its fixation. Site i = 28 has only one neighboring box with a high number of AA homozygotes (and q > 0.5), but this proves sufficient to fix the best-adapted genotype. Until approximately generation 50, sites i = 12 and i = 28 maintain the highest rates of fitness increase, exhibit frequencies closer to their final values (q = 1 or q = 0), and clearly support larger numbers of the corresponding genotype compared to their surroundings. As a result, migrants from these boxes are more genetically homogeneous than those from other boxes, and even the stochastic migration does not alter the overall evolutionary trend – homozygotes displace the less adapted heterozygotes.

On the migration balance S (i) k graphs (Fig. 5d), it can be observed that at the initial stages (t = 5), the distribution of both the direction and intensity of individual flows between sites appeared largely random and comparable across different genotypes. As spatial differentiation progresses and betteradapted individuals displace less adapted ones, homogeneous areas with the largest population sizes (i = 12 and i = 28) begin to contribute more significantly to migration than highly polymorphic areas. By the 60th generation, two monomorphic groups with opposite traits, AA and aa, reach their largest sizes (AA – 17 boxes, aa – 8 boxes) and come into contact. However, since they have by then accumulated a sufficient number of individuals and their population sizes prove to be comparable, the resulting migrant flows also become comparable, despite the 11 % difference in fitness. As a result, in the hybrid zones near sites numbered i = 6 and i = 16, two equally large streams of individuals with opposite genotypes converge, ensuring a non-zero number of heterozygotes in these boxes. The outflow from these boxes is much weaker and is barely sufficient to maintain a low level of polymorphism in their vicinity. However, it is these hybrid zones that slow down the flows of homozygous individuals of different forms, preventing the better-adapted AA genotype from achieving complete fixation throughout its range.

## Discussion

The verification of model (9) against the experimental data from Yu.P. Altukhov’s study on box populations of D. melanogaster, along with the analysis of scenarios underlying the formation of heterogeneous distributions of allele frequencies and population sizes, requires further clarification

First, it is necessary to discuss the reason for the pronounced differences in fitness observed among genotypes with different allele combinations of the α-Gdph enzyme, as revealed by estimates of the selection coefficients sk. It is quite plausible that the α-Gdph locus serves as a marker of disruptive selection operating within the system, acting not directly on the α-Gdph gene itself, but on closely linked adaptive genes. This may explain certain discrepancies between the observed and modeled distributions and frequency dynamics, since the overall adaptive effect and direction of selection – even for genes strongly linked to α-Gdph – are not simply additive. Instead, they result from more complex interactions, such as polygenic or complementary gene effects, epistasis, or multigene interaction.

Note that a significant difference in fitness is not a necessary condition for genetic divergence in model (1). It has been previously demonstrated that spatial differentiation can occur even with small differences in fitness. The degree of difference between genotypes, as well as the migration coefficient, determines the rate at which stable divergence is achieved, and the size of the resulting monomorphic subpopulations and hybrid zones (Kulakov, Frisman, 2025).

Despite the limitations noted above, the proposed model allows to analyze the processes that led to the primary genetic divergence observed in the experiment. It was found that the combined effect of genetic drift, density-dependent limitation, and gene flow – before the effective population size Ne and the minimum number of breeders N0 were reached – resulted in some boxes accidentally containing a higher number of less adapted aa individuals than the more adapted AA ones. As a result, subpopulations with even a slight deviation in allele frequencies from the theoretically expected values (typical for a local panmictic population) reached the highest average fitness and population growth rate earlier than others. As emigrants carry the allelic composition of their source subpopulation, clusters of boxes with either AA or aa genotypes form around these rapidly growing groups. Gradually, these genotypes displace the less-adapted heterozygous Aa individuals and occupy the largest number of sites. The interaction between the two migrant streams, carrying AA and aa genotypes, maintains a non-zero number of heterozygous individuals in certain boxes, creating hybrid zones. On the one hand, their presence preserves the genetic diversity of the entire metapopulation. On the other hand, these zones prevent the fittest individuals from occupying the entire range.

This evolutionary scenario can be considered universal for several reasons. The divergence of natural populations is always preceded by the emergence of mutants with a new trait in certain areas. For such a trait to become fixed, especially if it confers no significant immediate advantage, strong reproductive isolation from the parental population is required. This may be a case of disruptive selection, which is manifested not only in the reduced fitness of heterozygotes (hybrids) but also in positive assortative mating, which further diminishes the reproductive success of small hybrid populations. For instance, in the case of the hooded and carrion crow mentioned in the Introduction, the primary isolating mechanism appears to be based on mating preferences. For crows, plumage color is significantly associated with innate perception of potential partners, which substantially reduces the likelihood of mating between dissimilar morphs but allows for crossbreeding between already hybrid individuals or between hybrid and “pure” forms (Poelstra et al., 2014; Kryukov, 2019).

Unlike seasonal migration, the dispersal of individuals and colonization of new sites is a slow process that unfolds over multiple generations. Consequently, the remote parts of a new area will be inhabited only by the descendants of the original migrants. During this gradual expansion, individuals will inevitably interbreed with local populations. The model proposed in this paper demonstrates that such dispersal will inevitably cease if the recipient site is inhabited by individuals possessing a different trait than the migrants, due to potential selection against hybrids. In the case of crows, assortative mating will restrict interbreeding between the different morphs in newly colonized areas, thereby significantly reducing the likelihood of further expansion. In the ring populations’ system of Drosophila, the reduced fitness of heterozygotes decreases hybrid fertility and prevents their descendants from dispersing further. Consequently, for species where dispersal is a multigenerational process, hybrid zones act as significant barriers. They effectively impede the movement of individuals possessing one trait into areas occupied by individuals with another trait, without the need for those areas to be permanently settled, and with a high probability of producing hybrid offspring. If a more rapid dispersal mechanism is possible, this dynamic can change dramatically.

## Conclusion

The dynamic model proposed in this paper enables a detailed investigation of the mechanisms underlying primary genetic divergence. These mechanisms are attributed to differences in genotype fitness, settlement patterns, migration, and the formation of stable hybrid zones. The model demonstrates the possibility of reproductive isolation between different forms of diploid organisms, which arises not only from geographical isolation, habitat remoteness, or ecological specialization but also from hereditary mechanisms, genetic drift, gene flow, and selection against heterozygotes. This type of selection results in stable spatial genotype differentiation, maintained by hybrid zones that act as effective barriers to the introgression of divergent traits.

Thus, disruptive selection is demonstrated to play a crucial role – an effect that can be detected through certain marker genes but is not always apparent from external morphology. Consequently, it may be far more widespread in nature than previously believed.

## Conflict of interest

The authors declare no conflict of interest.
